# Cumulative live birth rates and birth outcomes after IVF/ICSI treatment cycles in young POSEIDON patients: A real-world study

**DOI:** 10.3389/fendo.2023.1107406

**Published:** 2023-03-30

**Authors:** Enqi Yan, Wenxuan Li, Huizi Jin, Mengya Zhao, Dan Chen, Xinyao Hu, Yifan Chu, Yaxin Guo, Lei Jin

**Affiliations:** Reproductive Medicine Center, Tongji Hospital, Tongji Medical College, Huazhong University of Science and Technology, Wuhan, China

**Keywords:** POSEIDON criteria, cumulative live birth rates, birth outcomes, low prognosis, IVF/ICSI

## Abstract

**Objective:**

The aim of this study was to describe the cumulative live birth rates (CLBRs) of young women with or without low prognosis according to the POSEIDON criteria after IVF/ICSI cycles and to investigate whether the diagnosis of low prognosis increases the risk of abnormal birth outcomes.

**Design:**

Retrospective study.

**Setting:**

A single reproductive medicine center.

**Population:**

From January 2016 to October 2020, there were 17,893 patients (<35 years) involved. After screening, 4,105 women were included in POSEIDON group 1, 1,375 women were included in POSEIDON group 3, and 11,876 women were defined as non-POSEIDON.

**Intervention(s):**

Baseline serum AMH level was measured on the D2–D3 of menstrual cycle before IVF/ICSI treatment.

**Main outcome measure(s):**

Cumulative live birth rate (CLBR), birth outcomes.

**Result(s):**

After four stimulation cycles, the CLBRs in POSEIDON group 1, POSEIDON group 3, and non-POSEIDON group reached 67.9% (95% CI, 66.5%–69.3%), 51.9% (95% CI, 49.2%–54.5%), and 79.6% (95% CI, 78.9%–80.3%), respectively. There was no difference in gestational age, preterm delivery, cesarean delivery, and low birth weight infants between the three groups, but macrosomia was significantly higher in non-POSEIDON group, after adjusting for maternal age and BMI.

**Conclusion(s):**

The POSEIDON group shows lower CLBRs than the non-POSEIDON group in young women, while the risk of abnormal birth outcomes in the POSEIDON group will not increase.

## Introduction

Infertility refers to the failure of establishing a clinical pregnancy after 1 year of regular, unprotected sexual intercourse, affecting 8%–12% couples of child-bearing ages worldwide (1). Thanks to the development of assisted reproductive technology (ART), the unwanted non-conception problem has been solved in more than 60% of young husbands and wives (2). As the most widely used technique of ART, *in vitro* fertilization (IVF) is a process of depletion, which means that in IVF procedure, qualitative and quantitative performance of ovarian reserve both counts (3). Patients with diminished ovarian reserve (DOR) and poor ovarian response (POR) may suffer from both a reduction in the number of eggs retrieved and ovarian quality (4). Practically, treating and consulting these patients are somewhat challenging because they usually need to receive more than one stimulation cycle before attaining a live birth that may bring about overwhelming monetary and time-consuming issues (5).

In ART cycle, female age is the utmost factor leading to success because aneuploidy rate in embryos increases when women age, and aneuploid embryos result in a higher risk of pregnancy loss or chromosomally abnormal pregnancy (6). Besides age, ovarian reserve test (ORT) before stimulation provides information on the remaining follicular pool and can predict the prognosis to ovarian stimulation. Reliable markers of ovarian reserve include FSH, anti-Müllerian hormone (AMH), and antral follicle counts (AFCs). AMH is more sensitive than FSH and is a frequently used measurement in extensive literatures (7). At the same time, the number of oocytes retrieved can be treated as a *post-hoc* test to determine the actual reactions to exogenous gonadotropins for the next cycle.

To better predict prognosis, POSEIDON (Patient-Oriented Strategies Encompassing Individualized Oocyte Number) criteria take into account the above aspects of quantity and quality (8, 9). Under this system, patients are divided into four groups. POSEIDON women are defined by age and ovarian reserve. POSEIDON groups 1 and 2 are the unexpected suboptimal or poor responders characterized by owning an adequate ovarian reserve but hypo-response to standard ovarian stimulation (≤9 eggs retrieved). POSEIDON groups 3 and 4 are the expected poor responders characterized by a decreased ovarian reserve. POSEIDON groups 1 and 3 are women who are younger than 35 years, and the other two groups are older than 35.

With the introduction of the concept “cumulative live birth rate (CLBR)” (10), the conventional evaluation of success in IVF transforms from taking single cycle into consideration solely to comprise the whole fresh and subsequent frozen–thawed transfers. Referencing the CLBR per woman helps physicians and patients make better treatment decisions. From current published data, we are informed that repeated stimulation provides much more benefit to young patients. In fact, a study performed by the researchers in our center showed that in women <38 years of age who are diagnosed with POR with Bologna criteria, the conservative and optimistic CLBRs after six treatment cycles are obviously superior to that of women of advanced age (11).

As for infertile women who are at risk of low prognosis, the most concern is the opportunity to eventually take home a healthy baby. For this consideration, not only the chance of giving birth but also the health of newborns is also noteworthy. Furthermore, IVF treatment is connected to a higher risk of abnormal perinatal outcomes compared to natural pregnancy and intrauterine insemination (12). A study conducted by Hu et al. (13) analyzed the perinatal outcomes in young patients diagnosed as DOR and found no increased risk of obstetrics and birth outcomes compared to non-DOR group when fresh cycles are performed. However, there is limited study focusing on birth outcomes of young POR and DOR patients when thawed frozen embryos are transplanted. Therefore, we conducted a retrospective study to provide these patients with more comprehensive information about pregnancy and delivery. We used the POSEIDON criteria to classify the studied patients. The case groups included POSEIDON groups 1 and 3, and the control group was the “non-POSEIDON group.” The objectives of the present study were twofold: (i) making a comparison of CLBRs between case and control groups and (ii) assessing the birth outcomes of the studied population.

## Materials and methods

### Study population and design

Our center started the long-term delivery follow-up in the year 2016. Therefore, we retrospectively analyzed the clinical data of patients who had their first ovarian stimulation at the Reproductive Medicine Center of Tongji Hospital during the period from January 2016 to October 2020. The subsequent cycles of the studied populations were included until either the first live birth achieved or they dropped out of the cohort, whichever came first. The follow-up continued until 31 October 2021. We used AMH value to determine the ovarian reserve, and it was measured within 1 year before IVF/ICSI stimulation. Patients with any of the following characteristics were excluded (1): donor oocytes received, (2) oocytes cryopreservation, (3) without detailed information on ovarian stimulation, and (4) using “non-standard” ovarian stimulation protocol in the first cycle. For birth outcomes analysis, we excluded the vanishing twin syndrome and multiple live births. That is, patients with singleton pregnancies were only considered.

Clinical pregnancy was determined by the ultrasonographic visualization of one or more gestational sacs. Live birth was defined as the birth of one or more live infant(s) after 28 weeks of gestation. This study was approved by the Institutional Review Board of Tongji Hospital, Tongji Medical College, Huazhong University of Science and Technology.

### The definition of young POSEIDON patients

We used POSEIDON criteria to categorize our studied subjects. Female age was recorded at the first stimulation cycle. We defined young patients as those with age <35 years. The study of Esteves et al. (14) found an equivalent effect of AMH and AFC to classify POSEIDON patients, and the two markers are both practicable. Hence, AMH was used as the ovarian reserve biomarker because it is a sensitive and relatively stable measurement between inter- and intra-cycle (7). POSEIDON group 1 included patients who had a normal ovarian reserve (AMH ≥1.2 ng/ml) but had an unexpected low response to standard ovarian stimulation in the first cycle (retrieved eggs ≤9). POSEIDON group 3 comprised of women with a diminished ovarian reserve (AMH <1.2 ng/ml). The control group was defined as “non-POSEIDON patients,” which included young women who had an optimal response (retrieved eggs >9) to ovarian stimulation with a normal ovarian reserve (AMH ≥1.2 ng/ml).

### IVF/ICSI procedures

Details about ovarian stimulation, egg retrieval, IVF/ICSI, embryo culture, morphological grading, vitrification cryopreservation and warming procedures, and embryo transfer have been described in previous research (11, 15–17). Standard ovarian stimulation treatment included GnRH agonist and GnRH antagonist protocols. GnRH agonist protocols included GnRH agonist long, GnRH agonist short, and depot GnRH agonist protocols. Ovarian stimulation regimen selection was based on female age, ovarian function, body mass index (BMI), and other characteristics by experienced physicians. During the procedure, administration dosage of gonadotropins was adjusted according to the response of stimulation for each patient. Once two to three leading oocytes reached a mean diameter of 14 mm by transvaginal ultrasound, recombinant hCG (250 mg; Ovidrel; Merck-Serono) was used to trigger ovulation. Oocytes were then fertilized through either conventional IVF or ICSI. D2 or D3 embryos were transferred freshly after egg retrieval. The surplus embryos that meet the frozen criteria were vitrified for later FET cycles. Embryos not considered to be frozen at the cleavage stage were cultured to day 5, 6, or 7 before vitrification. Some patients had all their surplus embryos cultured to the blastocyst stage before vitrifying. In circumstances not suitable for fresh ET, such as elevated E or P level, or inadequate uterine cavity, the freeze-all policy was implemented. Endometrial thickness (EMT) was assessed by ultrasound transvaginally. We recorded EMT at hCG day when patients received fresh embryos transplantation or when they had frozen–thawed embryos transfer (FET) with a natural or a stimulated endometrial preparation protocol. When the programmed cycles were performed in FET, we recorded EMT at the day of progesterone initiation. The maximum number of embryos transferred was two.

### Data collection and outcomes measurement

Sociodemographic data, IVF/ICSI data, pregnancy outcomes, and birth outcomes of the patients who meet the criteria were abstracted from the electronic medical record system in our hospital. Whether women did or did not give birth at Tongji, information on pregnancy and delivery was collected through telephone interviews by trained nurses at certain points before and after childbirth. The content of the follow-up interviews was described elsewhere (16). Seven women who had live births could not be contacted and were lost to follow-up. The main outcome of this study was the cumulative live birth rate (CLBR). The secondary endpoints were birth outcomes, which included gestational age, preterm birth, cesarean delivery, low birthweight, and macrosomia. Preterm birth was defined as a live birth before 37 weeks of gestation. Low birthweight was defined as the birthweight of a full-term delivered baby <2,500 g, and macrosomia referred to the birthweight of a newborn >4,000 g.

### Statistical analysis

Continuous variables are described as medians (first and third quartile) and were compared using Kruskal–Wallis or Mann–Whitney U test. Categorical variables were presented as frequency and were analyzed with Pearson chi-square or Fisher’s exact test (Bonferroni correction in *post-hoc* test). Pregnancy outcomes were described in each entire treatment cycle, and CLBR was shown in a conservative manner. The conservative CLBR was calculated as the live births of all cycles (including the exact treatment cycle being described and previous cycles before it) divided by the number of people involved in the first cycle and was also reported as ratios and 95% confidence intervals (CIs). Birth outcomes were compared among patients who had singleton live birth, no matter which treatment cycle it occurred. Multiple logistic regression was performed on birth outcomes analysis to eliminate the confounding factors including maternal age and BMI. All these calculations were analyzed by SPSS 26.0 (IBM, Chicago, IL). p-values were two-sided, and p<.05 was considered statistically significant.

## Results

### Patients and treatment characteristics

**Figure 1:**
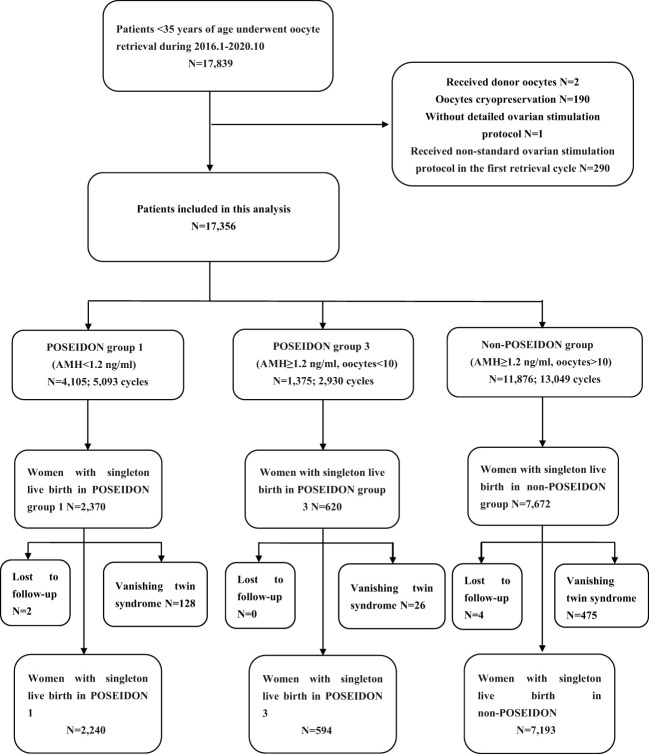
Flowchart of the participants.

As shown in **Figure 1**, a total of 17,839 patients under the age of 35 underwent their first oocyte retrieval at our center between January 2016 and October 2020. After the exclusion of patients who received donor oocytes, cryopreserved eggs, or without detailed ovarian stimulation protocol, a total of 17,356 patients were included in this study. Among them, 4,105 women of POSEIDON group 1 underwent 5,073 cycles of fresh ETs and 2,441 cycles of FETs. A total of 1,375 women of POSEIDON group 3 underwent 1,920 cycles of fresh ETs and 1,010 cycles of FETs, and 11,876 women of the non-POSEIDON group underwent 13,049 cycles of fresh ETs and 10,373 cycles of FETs.

**Table 1** shows the baseline characteristics of the patients included in this study. Women in the non-POSEIDON group were younger and had the lowest BMI and higher AMH and AFC values, implying superior ovarian reserve compared with POSEIDON groups 1 and 3. Women in POSEIDON group 3 had the lowest ovarian reserve presenting as the lowest AFC value and the largest proportion of women with AFC <5. Among all the causes of infertility, the most common cause in the three groups were pelvic and tubal factors, followed by male factor. Women from the non-POSEIDON group had the highest proportion of male factor, which led to the most frequent ICSI utilization in this group. In addition, non-POSEIDON women had the highest rate of polycystic ovary syndrome, which accounted for 15.5%. POSEIDON group 3 had highest proportion of endometriosis and prior ovarian surgery. In addition, there was no significant difference in the types of infertility among the three groups, and primary infertility accounted for >70%.

**Table 1:** Baseline characteristics.

	POSEIDON Group 1, n=4,105	POSEIDON Group 3, n=1,375	Non-POSEIDON Group, n=11,876	p-value
Characteristics				
Age (years)	30.0^#^ (28.0, 32.0)	30.0^#^ (28.0, 32.0)	29^#^ (27, 31)	<0.001*
BMI (kg/m^2^)	21.5^#^ (19.6, 23.8)	21.5^#^ (19.5, 23.8)	21.2 (19.5, 23.4)	0.001*
Infertility type				.533
Primary	2,960 (72.1)	984 (71.6)	8455 (71.2)	
Secondary	1,145 (27.9)	391 (28.4)	3,421 (28.8)	
Duration of infertility (years)	3.0 (2.0, 4.0)	2.0^#^ (1.0,4.0)	3.0 (2.0, 4.0)	0.001*
AMH (ng/mL)	3.1^#^ (2.0, 5.4)	0.8^#^ (0.5,1.0)	5.7 (3.7, 8.8)	<0.001*
AFC^&^	11.0^#^ (8.0, 15.0)	5.0^#^ (3.0,7.0)	16.0 (12.0, 22.0)	<0.001*
AFC<5 (n) ^&^	210^#^ (5.1)	580^#^ (42.2)	71 (0.6)	<0.001*
Infertility diagnosis				
Polycystic ovary syndrome	388^#^ (9.5)	4^#^ (0.3)	1,835 (15.5)	<0.001*
Pelvic and Tubal factors	2,172 (52.9)	628^#^ (45.7)	6,263 (52.7)	<0.001*
Endometriosis	461^#^ (11.2)	268^#^ (19.5)	878 (7.4)	<0.001*
Male factor	1,362^#^ (33.2)	363^#^ (26.4)	4,363 (36.7)	<0.001*
Uterine factor	589 (14.3)	205 (14.9)	1626 (13.7)	0.323
Chromosome abnormality	133^#^ (3.2)	44 (3.2)	509 (4.3)	0.004*
Prior ovarian surgery	73^#^ (1.8)	48^#^ (3.5)	120 (1.0)	<0.001*

BMI, body mass index; AMH anti-Müllerian hormone; AFC, antral follicle counts.

^#^Pairwise comparison between POSEIDON and non-POSEIDON group indicates significant difference using Bonferroni multiple comparison test.

^&^Based on 17,332 patients, 0.14% data are missing.

*p<0.05.

The IVF/ICSI outcomes and transplantation outcomes are presented in **Table 2**. Regarding the ovarian stimulation protocol, the most common choice in POSEIDON groups 1 and 3 was GnRH antagonist, accounting for 40.0% and 49.9%, respectively. The depot GnRH agonist regimen was more frequently received by women in the non-POSEIDON group, reaching 54.5% in non-POSEIDON women. After egg retrieval, non-POSEIDON group had the highest number of oocytes, available embryos, available blastocysts, fertilization rate, and blastocyst formation rate, while POSEIDON group 3 was on the contrary. For embryo transfer, women in POSEIDON group 1 had the least population of freezing all their embryos, and 52.7% of cycles were performed with fresh cycles only. Among all embryo transfer cycles, the average number of embryos transferred was one in POSEIDON group 3 and two in POSEIDON group 1 and non-POSEIDON group. The majority of women in POSEIDON groups 1 and 3 had transplanted cleavage stage embryos, while more than half of the non-POSEIDON women had blastocysts transferred. In all the three groups, only a few women chose to transfer morula embryos.

**Table 2:** Treatment characteristics.

	POSEIDON Group 1, n=4,105	POSEIDON Group 3, n=1,375	Non-POSEIDON Group, n=11,876	p-value
Treatment characteristics				
Stimulation cycles	5,093	1,920	13,049	–
Protocol				<0.001*
GnRH agonist long	1,021^#^ (20.0)	97^#^ (5.1)	3,380 (25.9)	
GnRH agonist short	0 (0.0)	1 (0.1)	1 (0.0)	
GnRH antagonist	2,035^#^ (40.0)	959^#^ (49.9)	2,475 (19.0)	
Depot GnRH agonist	1,826^#^ (35.0)	92^#^ (4.8)	7,113 (54.5)	
Others	211^#^ (4.1)	711^#^ (40.2)	80 (0.6)	
Type of gonadotrophins				<0.001*
FSH	699^#^ (13.7)	225^#^ (11.7)	3,233 (24.8)	
HMG	40^#^ (0.8)	136^#^ (7.1)	18 (0.1)	
FSH+LH(HMG)	4,350^#^ (85.4)	1,512^#^ (78.8)	9,798 (75.1)	
No use	4^#^ (0.1)	47^#^ (2.4)	0 (0.0)	
Total gonadotropin dose (IU)	2,475^#^ (1,987.5, 3,000)	2,700^#^ (2,100, 3,300)	2,025 (1,575, 2,625)	<0.001*
Stimulation duration (days)	10.0^#^ (9.0,11.0)	9.0^#^ (8.0,10.0)	10.0 (9.0,11.0)	<0.001*
Follicles≥14mm at hCG administration	8.0^#^ (6.0,10.0)	4.0^#^ (3.0,6.0)	13.0 (10.0,16.0)	<0.001*
Peak E2 value (pg/ml)^&1^	1,738.0^#^ (1,245.0, 2,483.0)	1,077.5^#^ (718.0,1,602.8)	3,000.0 (2,184.0, 4,581.0)	<0.001*
Endometrial thickness (mm)^&2^	11.4^#^ (9.9,13.2)	9.9^#^ (8.0,11.7)	11.8 (10.2,13.5)	<0.001*
Number of fresh cycles				<0.001*
IVF cycles	3,301^#^ (64.8)	1,366^#^ (71.1)	8,145 (62.4)	
ICSI cycles	1,498^#^ (29.4)	473^#^ (24.6)	4,153 (31.8)	
IVF+ICSI cycles	294 (5.8)	81^#^ (4.2)	751 (5.8)	
PGT	78^#^ (1.5)	30^#^ (1.6)	333 (2.6)	<0.001*
Number of oocyte retrieval cycles	15^#^ (0.3)	64^#^ (3.3)	0 (0.0)	<0.001*
Number of oocytes retrieved	7.0^#^ (5.0,9.0)	4.0^#^ (2.0,7.0)	15.0 (12.0,20.0)	<0.001*
Number of metaphase II oocytes	6.0^#^ (4.5,8.0)	4.0^#^ (2.0,6.0)	13.0 (10.0,17.0)	<0.001*
Fertilization rate (%)	21,304^#^ (67.3)	5,270^#^ (65.3)	128,541 (69.1)	<0.001*
Number of fertilized oocytes (2PN)	4.0^#^ (2.0,6.0)	2.0^#^ (1.0,4.0)	9.0 (7.0,12.0)	<0.001*
Number of obtained embryos	2.0^#^ (2.0,3.0)	2.0^#^ (1.0,3.0)	4.0 (3.0,6.0)	<0.001*
Blastocyst formation rate (%)	9,197^#^ (62.9)	1,881^#^ (60.4)	75,918^#^ (67.7)	<0.001*
Number of available blastocyst	1.0^#^ (0.0,2.0)	0.0^#^ (0.0,1.0)	3.0 (1.0,6.0)	<0.001*
Number of available embryos in FET cycles	1.0^#^ (0.0,2.0)	1.0^#^ (0.0,2.0)	3.0 (2.0,6.0)	<0.001*
Elective embryo freezing	907^#^ (17.9)	690 (35.9)	4,801 (36.8)	<0.001*
Number of embryos transferred	2.0^#^ (1.0,2.0)	1.0^#^ (0.0,2.0)	2.0 (1.0,2.0)	<0.001*
Type of transfer, n (%)				<0.001*
Fresh only	2,682^#^ (52.7)	606^#^ (31.6)	5,127 (39.3)	
FET only	819^#^ (16.1)	595 (31.0)	4,322 (33.1)	
Fresh and FET	1,099^#^ (21.6)	224^#^ (11.7)	3,118 (23.9)	
No transfer	493^#^ (9.7)	495^#^ (25.8)	482 (3.7)	
Type of embryo transplanted				<0.001*
Cleavage embryo	6,144^#^ (72.5)	1,923^#^ (77.8)	11,708 (45.7)	
Morula embryo	2^#^ (0.0)	3^#^ (0.1)	0 (0.0)	
Blastocyst	2,334^#^ (27.5)	547^#^ (22.1)	13,912 (54.3)	
Pregnancy outcomes				
Singleton live birth	2,370^#^ (57.7)	620^#^ (45.1)	7,672 (64.6)	<0.001*
Vanishing twin syndrome	132^#^ (3.2)	26^#^ (1.9)	489 (4.1)	<0.001*
Multiple live births	418^#^ (10.2)	93^#^ (6.8)	1,780 (15.0)	<0.001*

GnRH, gonadotropin-releasing hormone; FSH, follicle-stimulating hormone; HMG, human menopausal gonadotropin; LH, luteinizing hormone; hCG, human chorionic gonadotropin; IVF, *in vitro* fertilization; ICSI, intracytoplasmic sperm injection; PGT, preimplantation genetic testing; FET, frozen–thawed embryo transfer.

^#^Pairwise comparison between POSEIDON and non-POSEIDON group indicates significant difference using Bonferroni multiple comparison test.

^&1^Based on 19,970 patients, 0.46% data are missing.

^&2^Based on 20,042 patients, 0.10% data are missing.

*p<0.05.

### Cumulative live birth rate

**Figure 2:**
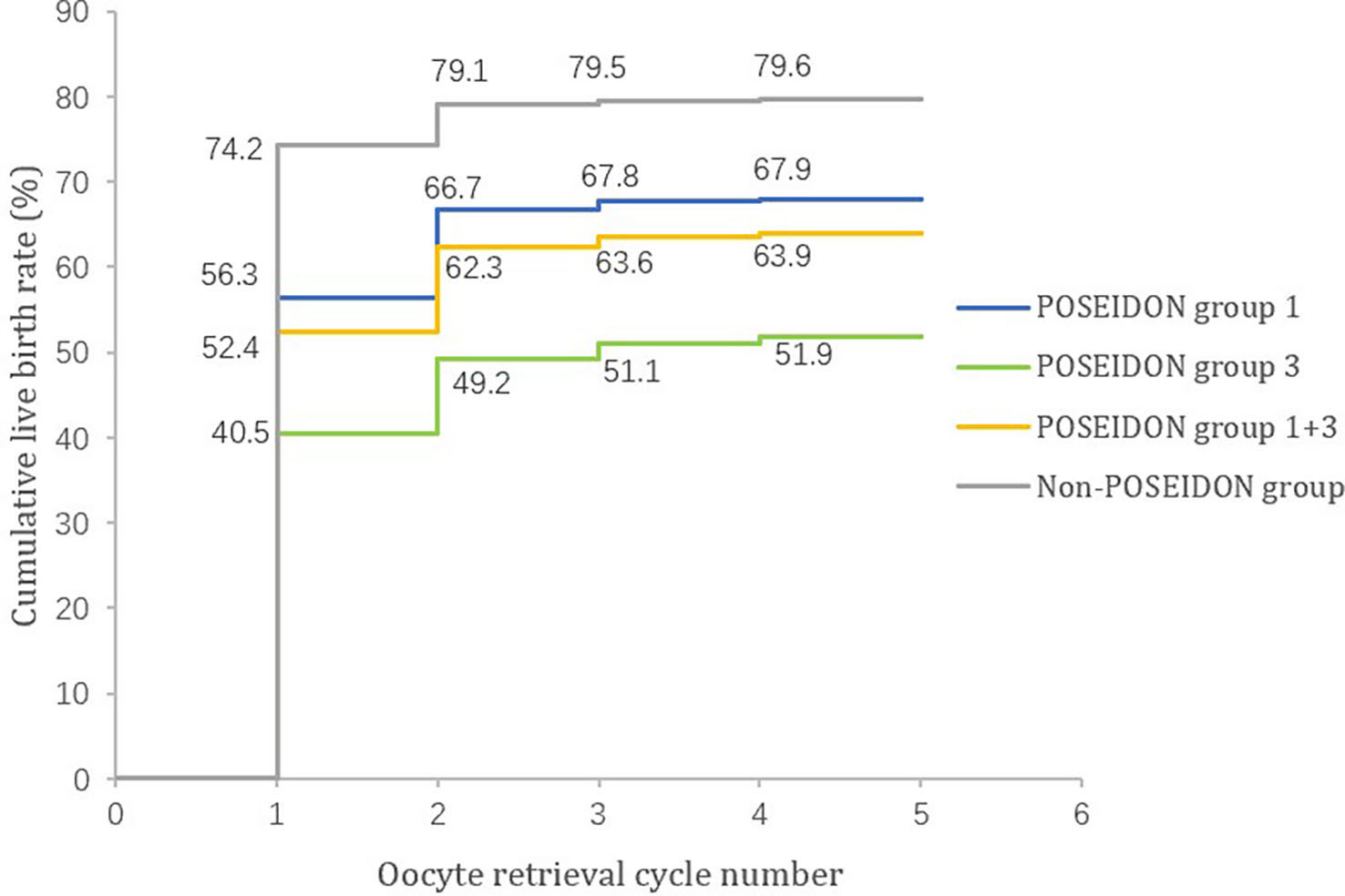
Cumulative live birth rates of young patients in POSEIDON and non-POSEIDON groups.

**Table 2** shows the pregnancy outcomes in the three groups. Singleton live birth, multiple live births, and vanishing twin syndromes were higher in the non-POSEIDON group than that in the other two groups. The transplantation, pregnancy, and live birth outcomes of each oocyte retrieval cycle of the three groups are shown in **Table 3**. The conservative CLBRs of each POSEIDON group are shown in **Figure 2**. After three to four treatment cycles, the live birth curves of the three groups tended not to ascend anymore. After four cycles of oocyte retrieval, the CLBRs in the three groups reached 67.9% (95% CI, 66.5%–69.3%), 51.9% (95% CI, 49.2%–54.5%), and 79.6% (95% CI, 78.9%–80.3%), respectively.

**Table 3:** Cumulative live birth rates in different groups.

Cycle	Cycle cohort (n)	Retrieval (n)	Transfer (n)	Pregnancies (n)	Live births (n)	Conservative LBR (95%CI)
POSEIDON group 1						
1	4,105	4,095	3,746	2,616	2,312	56.3 (54.8, 57.8)
2	845	842	746	494	425	66.7 (65.2, 68.1)
3	115	114	89	56	46	67.8 (66.4, 69.2)
≥4	28	27	19	6	5	67.9 (66.5, 69.3)
POSEIDON group 3						
1	1,375	1,335	1,059	647	557	40.5 (37.9, 43.1)
2	401	390	285	149	119	49.2 (46.5, 51.8)
3	98	93	57	31	27	51.1 (48.5, 53.8)
≥4	46	38	24	12	10	51.9 (49.2, 54.5)
Non-POSEIDON group						
1	11,876	11,876	11,502	9,678	8,816	74.2 (73.4, 75.0)
2	1,051	1,051	957	667	582	79.1 (78.4, 79.9)
3	108	108	96	60	49	79.5 (78.8, 80.3)
≥4	14	14	12	6	4	79.6 (78.9, 80.3)

LBR, live birth rate.

### Birth outcomes

After excluding the vanishing twin syndrome and the cases lost to follow-up, the information of 2,240, 594, and 7,193 patients with singleton live birth is shown in **Table 4**. Non-POSEIDON women who had singleton live birth remained the youngest. At the time of each live birth, the embryos of women in the POSEIDON group were mainly from fresh cycles, while those of non-POSEIDON women were mainly from FET cycles. For birth outcomes, cesarean delivery rates were high in all the three groups, reaching approximately 80%. There was no significant difference in preterm delivery and low birth weight infants between the three groups, but macrosomia occurred in 5.1% in non-POSEIDON women, which was significantly higher than that in POSEIDON group 1 (0.5%) and POSEIDON group 3 (0.8%). After adjusting for maternal age and BMI, the outcomes remained unchanged (**Supplementary Table 1**).

**Table 4:** Baseline characteristic and birth outcomes of women with singleton live birth.

	POSEIDON Group 1, n=2,240	POSEIDON Group 3, n=594	Non-POSEIDON Group, n=7,193	p-value
Characteristics				
Maternal age (years)	30.0^#^ (28.0, 32.0)	30.0^#^ (28.0, 32.0)	29.0 (27.0, 31.0)	<0.001*
Maternal BMI (kg/m^2^)	21.5^#^ (19.6, 23.9)	21.5 (19.5, 23.8)	21.2 (19.5, 23.4)	<0.001*
Endometrial thickness^&^ (mm)	10.6^#^ (9.2, 12.6)	10.0 (9.0, 11.6)	10.0 (8.9, 12.0)	<0.001*
Infertility type				0.831
Primary infertility	1,625 (72.5)	424 (71.4)	5,184 (72.1)	
Secondary infertility	615 (27.5)	170 (28.6)	2,009 (27.9)	
Duration of infertility (years)	3.0 (2.0, 4.0)	2.0^#^ (1.0, 4.0)	3.0 (2.0, 4.0)	
Type of transfer				<0.001*
Fresh cycles	1,361^#^ (60.8)	299^#^ (50.3)	3,213 (44.7)	
FET cycles	879^#^ (39.2)	295^#^ (49.7)	3,980 (55.3)	
Number of embryos transferred	1.0^#^ (1.0, 2.0)	1.0 (1.0, 2.0)	1.0 (1.0, 1.0)	<0.001*
Embryo stage				<0.001*
Cleavage embryo	1,459^#^ (65.1)	412^#^ (69.4)	2,837 (39.4)	
Morula embryo	1 (0.0)	1^#^ (0.2)	0 (0.0)	
Blastocyst	780^#^ (34.8)	181^#^ (30.5)	4,356 (60.6)	
Egg retrieval cycle				<0.001*
1	1,859^#^ (83.0)	462^#^ (77.8)	6,697 (93.1)	
2	337^#^ (15.0)	99^#^ (16.7)	453 (6.3)	
≥3	44^#^ (2.0)	33^#^ (5.6)	43 (0.6)	
Birth outcomes				
Gestational age (days)	273^#^ (267, 277)	273 (268, 277)	273 (268, 278)	0.090
Preterm delivery, <37 weeks	170 (7.6)	48 (8.1)	610 (8.5)	0.403
Cesarean delivery	1,792 (80.0)	476 (80.1)	5,659 (78.7)	0.323
Low birth weight, <2,500 g	22 (1.0)	9 (1.5)	58 (0.8)	0.180
Macrosomia, >40.00 g	12^#^ (0.5)	5^#^ (0.8)	368 (5.1)	<0.001*

BMI, body mass index; FET, frozen–thawed embryo transfer.

^#^Pairwise comparison between POSEIDON and non-POSEIDON group indicates significant difference using Bonferroni multiple comparison test.

^&^Endometrial thickness at the exact transfer cycle; 0.04% data are missing, based on 10,023 patients.

*p<0.05.

## Discussion

To the best of our knowledge, this is the first study to discuss the CLBRs together with the subsequent birth outcomes of young women with low prognosis and the normal prognosis after IVF/ICSI treatments. In the present research, we took into account the conservative CLBRs of our studied population, trying to show the real-world condition. Of the young patients, 31.6% were included in the POSEIDON groups, and the rest of them were categorized into the non-POSEIDON group. In our cohort, patients benefited from the first two treatment cycles, and the plateau appeared in the third cycle. After four oocyte retrieved cycles, the conservative CLBRs of the POSEIDON groups 1 and 3 reached 67.9% and 51.9%, respectively, and 79.6% for the non-POSEIDON group. As for birth outcomes, the three groups showed no difference in gestational age, preterm delivery, cesarean delivery, and low birth weight of the newborns when singleton live birth occurred. However, the non-POSEIDON patients showed a higher rate of macrosomia, and the difference remains significant after adjusting for maternal age and BMI.

Both the egg quality and quantity play an important role in ART success. Previous PGT-A data showed a parallel euploid embryo rate between young POSEIDON patients and their counterparts (18, 19). We confined our studied subjects to young patients and put more emphasis on the significance of quantitative aspect in ART success. The average number of oocytes retrieved in normal responders was 1.75× higher than that in POSEIDON group 1 and 3.75× higher than that in group 3. The subsequent embryos obtained and cryopreserved in non-POSEIDON patients were therefore the highest, so as the transferred cycles and CLBRs.

According to the published data, we are informed that CLBRs of patients with low prognosis received extensive attention (4, 5, 20–23). Studies report the CLBR of one whole aspiration IVF/ICSI cycle or CLBRs after several treatment cycles. However, definitions of POSEIDON patients in these studies are varied, so it is hard to make a comparison. Esteves et al. (4) published the first multicenter study to assess the CLBR of POSEIDON groups after one treatment cycle. They used AFC to be the hallmark and reported a CLBR of POSEIDON groups 1 and 3 and non-POSEIDON group as 45.7%, 29.4%, and 50.6%, respectively. Reporting CLBR is meaningful because the oocyte number is a robust indicator of live birth and CLBR takes into consideration the fresh cycle and all subsequent thawed frozen cycles (24). However, low responders often require more than one treatment cycle before success. In this case, reporting CLBRs after repetitive cycles may be of more significance than the CLBR after only one aspiration cycle. Abdullah et al. (5) reported similar CLBRs of young POSEIDON patients classified by AMH level and AFC to ours (77.3% for POSEIDON group 1 and 51.4% for group 3). The largest sample study of Li et al. (21) on Chinese patients is informative and representative. They conducted a study with 19,781 POSEIDON patients and showed that, after more than six cycles, the conservative CLBRs of young POSEIDON patients reached 66.13% and 29.76%. However, they lacked the results of control group.

Managing women with low prognosis is difficult. In clinical practice, reproductive experts must treat them based on their individual characteristics. There are many factors influencing the ART success for POSEIDON patients such as female age, BMI, infertility duration, treatment protocol, and baseline FSH (21). Of all these factors, ovarian stimulation protocols are of great clinical importance. Data from our center showed that in more than 3,000 POR patients, GnRH antagonist and progestin-primed ovarian stimulation (PPOS) protocols are more effective in improving live birth rate compared to GnRH agonist protocol (11), while among all the treatment protocols, natural cycle is of the least help. Zhang et al. (25) reported a higher CLBR after using GnRH antagonist than using PPOS protocol in all POSEIDON patients. More future studies should be performed to figure out the effect of the different protocols on the low responders.

For birth outcomes analysis, POSEIDON group 3 is associated with the highest proportion of endometriosis, which is reported to increase the risk of preterm delivery, caesarean delivery, and delivery of a small-for-gestational-age (SGA) infant (26). However, our findings did not show any difference in the POSEIDON group 3 compared to the other two groups. In our data, we found that the prevalence of macrosomia in the non-POSEIDON group was significantly higher. The difference may be caused by the higher PCOS rate, which increases the adverse neonatal outcomes such as preterm birth, delivery of a large-for-gestational-age (LGA) baby, and a low Apgar score (<7) (27). The adverse birth outcomes resulted from various factors, and what our study did was to report the real situation. Well-organized prospective studies are warranted to clarify this finding.

This study has some limitations. First of all, many patients in our study dropped out of the cohort without having a live birth. The reasons for not resuming treatment may be the financial burdens, psychological pressure, or medical advice from physicians to discontinue. Second, we only calculated the conservative CLBRs, but the condition may be superior because, according to the follow-up reviews, we were aware that some of the patients went to another center to continue their therapy. Third, due to the retrospective nature, we were unable to provide the situations of the pregnant mothers during the perinatal period. In addition, we were unable to provide the smoking status of the patients and the weight gain during pregnancy. Fourth, we only included the young POSEIDON groups in this research. Several previous studies did not set a control group, and among the research that took the non-POSEIDON group as the control group, a limited of them distinguish the patients according to age. Our study confirmed that the ovarian quantity may be the decisive factor in predicting success for young infertile women. We will further discuss the outcomes of elder POSEIDON patients in the near future.

## Conclusion

In conclusion, this long-term follow-up on pregnancy and delivery of young POSEIDON patients provides comprehensive information. It showed that after repeated ovarian stimulation, low responders can obtain more than 50% of live births. Diagnosing as a low responder did not increase the risk of abnormal birth outcomes, and normal responders may be associated with higher risk of delivering a macrosomia.

## Data availability statement

The raw data supporting the conclusions of this article will be made available by the authors, without undue reservation. 

## Ethics statement

The studies involving human participants were reviewed and approved by The Institutional Review Board of Tongji Hospital, Tongji Medical College, Huazhong University of Science and Technology. Written informed consent for participation was not required for this study in accordance with the national legislation and the institutional requirements.

## Author contributions

EY, YG, and LJ conceived of the study and participated in its design. EY and WL wrote the paper. WL and HJ analyzed the data. MZ, DC, XH, and YC collected the data. All co-workers have seen and agreed with the contents of the manuscript. All authors contributed to the article and approved the submitted version. 
