# Supplementary material for: Cumulative live birth rates and birth outcomes after IVF/ICSI treatment cycles in young POSEIDON patients: A real-world study
Source: Front Endocrinol (Lausanne). 2023 Mar 30;14:1107406. doi: 10.3389/fendo.2023.1107406 (PMC10098357; doi:10.3389/fendo.2023.1107406)
Supplement: Supplementary file 1 [file Table_1.docx]

**Supplemental table 1. Birth outcomes after multiple logistic regression.**

|  | **POSEIDON group 1 to non-POSEIDON group (Reference)** | | **POSEIDON group 3 to non-POSEIDON group** | |
| --- | --- | --- | --- | --- |
| **Birth outcomes** | **AOR** | **Adjusted P value** | **AOR** | **Adjusted P value** |
| Gestational age | 0.074 (-0.046, 0.195) | 0.227 | 0.124 (-0.299, 0.548) | 0.565 |
| Preterm delivery, <37 wk | 0.848 (0.710, 1.075) | 0.070 | 0.887 (0.651, 1.207) | 0.446 |
| Cesarean delivery | 1.031 (0.915, 1.161) | 0.620 | 1.027 (0.831, 1.269) | 0.805 |
| Low birth weight, <2,500 g | 1.257 (0.766, 2.063) | 0.365 | 1.944 (0.953, 3.964) | 0.068 |
| Macrosomia, >40,00 g | 0.093 (0.052, 0.166) | <0.001* | 0.148 (0.061, 0.360) | <0.001* |

*AOR* adjusted odds ratio

AOR, 95% CI, and Adjusted P value of gestational age was based on linear regression after adjusting for maternal age and maternal BMI. AORs, 95% CIs, and Adjusted P values of preterm delivery, cesarean delivery, low birth weight, and macrosomia were based on multiple logistic regression model after adjusting for maternal age and maternal BMI.

*P<0.05
